# Effect of long-term azithromycin treatment on gut microbial diversity in children and adolescents with HIV-associated chronic lung disease

**DOI:** 10.1016/j.ebiom.2025.105832

**Published:** 2025-07-05

**Authors:** Trym Thune Flygel, Ahmed Bargheet, Regina Esinam Abotsi, Shantelle Claassen-Weitz, Victoria Simms, Erik Hjerde, Kilaza Samson Mwaikono, Grace Mchugh, Dan Hameiri-Bowen, Veronika Kuchařová Pettersen, Rashida Abbas Ferrand, Mark Nicol, Jorunn Pauline Cavanagh, Trond Flaegstad, Evgeniya Sovershaeva

**Affiliations:** a Research Group of Child and Adolescent Health, Department of Clinical Medicine, Faculty of Health Sciences, UiT The Arctic University of Norway, Tromsø, Norway; b Department of Paediatrics, University Hospital of North Norway, Tromsø, Norway; c Host-Microbe Interaction Research Group, Department of Medical Biology, UiT The Arctic University of Norway, Tromsø, Norway; d Department of Molecular and Cell Biology & Institute of Infectious Diseases and Molecular Medicine, University of Cape Town, Cape Town, South Africa; e Department of Pharmaceutical Microbiology, School of Pharmacy, University of Health and Allied Sciences, Volta, Ghana; f Division of Medical Microbiology, University of Cape Town, Cape Town, South Africa; g MRC International Statistics and Epidemiology Group, Faculty of Epidemiology and Population Health, London School of Hygiene and Tropical Medicine, London, United Kingdom; h Department of Chemistry, Norstruct, UiT The Arctic University of Norway, Tromsø, Norway; i Computational Biology Group and H3ABioNet, Department of Integrative Biomedical Sciences, University of Cape Town, Cape Town, South Africa; j Department of Science and Laboratory Technology, Dar es Salaam Institute of Technology, Tanzania; k Biomedical Research and Training Institute, Harare, Zimbabwe; l Nuffield Department of Medicine, University of Oxford, Oxford, United Kingdom; m Department of Clinical Research, Faculty of Infectious and Tropical Diseases, London School of Hygiene and Tropical Medicine, London, United Kingdom; n Marshall Centre, Division of Infection and Immunity, School of Biomedical Sciences, University of Western Australia, Perth, Australia; o Department of Microbiology and Infection Control, Akershus University Hospital, Nordbyhagen, Norway

**Keywords:** HIV, Gastrointestinal microbiome, Southern Africa, Chronic lung disease, Azithromycin

## Abstract

**Background:**

HIV-associated chronic lung disease (HCLD) is common in children and adolescents growing up with HIV. The use of azithromycin (AZM) reduces the rate of acute respiratory exacerbations in this population, however, impact of this treatment on the gut microbiota and associations with blood-derived inflammatory markers have not been studied.

**Methods:**

Children and adolescents with HCLD in Harare, Zimbabwe and Blantyre, Malawi were recruited in a double-blind, placebo-controlled trial of once-weekly AZM or placebo for 48 weeks (BREATHE trial, NCT02426112). Rectal swabs were collected at inclusion (N = 346), 48 weeks (treatment end, N = 313), and 72 weeks (six months after treatment cessation, N = 244). The bacterial composition of fecal swabs was determined using 16S rRNA gene sequencing. Plasma biomarkers at inclusion and 48 weeks were measured using Luminex multiplex bead assay.

**Findings:**

At 48 weeks, bacterial α-diversity was significantly lower in the AZM group, with 27 bacterial genera showing differential abundance between the study groups. The placebo group exhibited higher interconnectivity between bacterial genera at 48 weeks compared to the AZM group. Correlations between the top seven differentially abundant genera and biomarkers observed at inclusion were no longer significant at 48 weeks in both groups. Depletion of *Campylobacter* persisted for six months after cessation of AZM treatment.

**Interpretation:**

Long-term AZM treatment in HCLD patients affects their gut bacterial composition at least 6 months after its cessation. The consequences of reduced bacterial diversity, such as altered interaction with the immune system and risk of resistance, need further investigation to understand how to optimise gut health during long-term antibiotic treatments.

**Funding:**

The study was funded by the 10.13039/501100005416Norwegian Research Council and Helse Nord (HNF 1387-17).

**Research in context**

**Evidence before this study**

The era of antiretroviral therapy is characterised by a growing burden of chronic comorbidities among HIV-infected individuals. Studies conducted in Sub-Saharan Africa indicate that children and adolescents with HIV are at increased risk of chronic lung complications. At present, no clinical guidelines are available for the management of HIV-associated chronic lung disease (HCLD). We recently conducted a randomised controlled trial (RCT), which demonstrated that long-term treatment with azithromycin (AZM) reduces the rate of acute respiratory exacerbations in this population. Despite the potential clinical benefits, long-term use of AZM is likely to disrupt the gut microbiome.

We searched PubMed with combinations of terms “HIV”, “gut microbiome”, “lung disease”, and “antibiotics” for articles published in English up to March 2025. We found evidence that HIV-infected individuals have decreased α-diversity of the gut microbiome, a decreased abundance of commensal bacteria, and enrichment in potentially pathogenic taxa. However, no studies so far investigated the impact of antibiotic treatment in HIV-infected individuals with chronic lung diseases.

**Added value of this study**

In this study, we investigated the impact of AZM treatment on gut bacterial composition, diversity, and community interactions in children and adolescents with HCLD. We used data collected as a part of the double-blinded, randomised, placebo-controlled trial BREATHE. In this trial, weekly AZM in weight-adjusted doses was given to children and adolescents aged 6–19 years with HCLD in Harare, Zimbabwe, and Blantyre, Malawi, for 48 weeks (BREATHE trial, clinicaltrials.gov identifier NCT02426112). We found that long-term AZM treatment reduced gut microbiome α-diversity in individuals with HCLD. Most changes in microbial composition after AZM treatment were transient, though β-diversity remained significantly different between the study groups six months after the cessation of the study drug.

Our study is one of the largest to assess the gut microbiome composition in HIV-infected children with chronic lung disease in Sub-Saharan Africa. So far, no studies have addressed the impact of prolonged antibiotic treatment on gut microbiome in this population. Access to comprehensive clinical data allowed us to study potential interactions between the gut microbiome and systemic plasma biomarkers.

**Implications of all the available evidence**

HIV infection is related to gut microbial dysbiosis, and previous studies have shown that antiretroviral therapy only partially restores the gut microbiota towards that of healthy individuals. Our study demonstrates that long-term treatment with AZM in individuals with HCLD leads to further deterioration of the gut microbiome, questioning the utility of prophylactic antibiotic administration in this population group. The long-term consequences of reduced gut microbial diversity and altered interaction with the host immune response, as well as the potential for an increase in antimicrobial resistance, should be considered when prescribing prophylactic antibiotics for individuals with HCLD.

## Introduction

The majority of CD4^+^ T-cells reside in gut-associated lymphatic tissue,^1^ which is one of the earliest sites of HIV infection. The infection results in CD4^+^ T-cell depletion, in addition to the impairment of the gastrointestinal barrier and consequent microbial translocation. Several studies have also reported alterations in the gut microbial composition of individuals with HIV.^2^ A dysbiotic gut microbiota and microbial translocation may contribute to systemic immune activation that occurs in HIV infection, leading to chronic inflammation and increased risk of non-infectious HIV-related complications, including heart and lung disease.^3^, ^4^, ^5^, ^6^ Previous studies have shown that people with HIV have a less favourable gut microbiota characterised by lower bacterial diversity, including loss of protective commensal bacteria (e.g., *Bifidobacteriales* and *Lactobacillales*) and a high level of proinflammatory bacteria such as *Enterobacterale*s.^7^ Further, these changes seem to persist after initiation of antiretroviral therapy (ART).^7^ However, it has also been suggested that ART can help restore the gut microbiota towards that of HIV-negative people.^8^^,^^9^

HIV-associated chronic lung disease (HCLD) is a common comorbidity among children and adolescents with HIV.^10^ Studies have shown that long-term azithromycin (AZM) treatment may improve lung function, reduce the number of infectious respiratory exacerbations, and increase survival in various chronic lung diseases, including cystic fibrosis, obliterative bronchiolitis, and non-cystic fibrosis bronchiectasis.^11^^,^^12^ Likely mechanisms include the antimicrobial, anti-inflammatory, and immunological effects of macrolides, an antibiotic class including AZM.^12^ Further, AZM is a commonly used antibiotic against acute respiratory tract infections. We have recently conducted a multi-site phase three randomised trial in children and adolescents with HCLD where AZM use was associated with a reduced number of acute respiratory exacerbations and hospitalisations.^13^

Despite these benefits, antibiotic use leads to dysbiosis of the gut microbiota,^14^^,^^15^ reducing the overall bacterial diversity as well as the abundance of antibiotic-sensitive taxa, many of which are commensal bacteria with various health-protective functions.^16^ The changes in the gut microbiota following antibiotic exposure can persist for years.^17^ Genera found to decrease after macrolide exposure include *Bifidobacterium*, *Campylobacter*, and *Lactobacillus*, whereas *Klebsiella*, *Eggerthella*, *Blautia*, *Dorea*, and *Pseudomonas* have been described to increase in relative abundance.^18^, ^19^, ^20^, ^21^ Given the fact that individuals with HIV often exhibit similar microbial alterations, the use of macrolides may further aggravate the shift in the microbial composition. Most studies report a normalisation of aerobic bacteria three weeks after cessation of macrolide treatment, whereas changes in anaerobic flora persist longer.^17^ Also problematic is the link between AZM use and an increased resistance to macrolides and other antibiotic classes.^22^ We have previously demonstrated an increase in AZM, tetracycline, and clindamycin resistance in *Staphylococcus aureus* from sputum samples of children with HCLD, persisting six months after completion of AZM treatment.^23^

Several studies have investigated the effect of long-term antibiotic treatment on the gut microbiota in children, but there are few studies in the context of HIV and HIV-associated comorbidities. This is of particular interest, since in people with HIV a dysbiotic gut microbiota has been reported, irrespective of antibiotic exposure.^6^^,^^8^ Understanding how AZM affects the already altered gut microbiota in children with HCLD will give insight into the pathogenesis of HCLD and guide treatment strategies in the future.

This study aimed to investigate the changes in gut bacterial diversity and composition in HIV-infected children and adolescents with HCLD receiving long-term AZM treatment. We analysed how AZM treatment affected the gut bacterial communities, their interactions, and the persistence of these changes six months after cessation of the antibiotic therapy. In addition, correlation analysis between gut microbiota and systemic plasma biomarkers was performed in order to study the interplay between gut microbiota and chronic inflammation.

## Methods

### Study participants

**Fig. 1:**
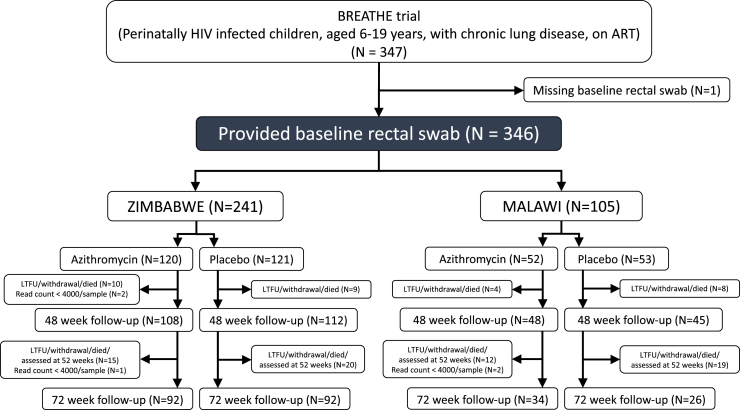
**Study flow chart.** Abbreviations: ART, antiretroviral therapy; LTFU, lost to follow-up.

This study was nested within the BREATHE trial; a double blinded, randomised, placebo-controlled trial of weekly weight-adjusted AZM (child's weight-to-AZM ratio: 10–19.9 kg, 250 mg; 20–29.9 kg, 500 mg; 30–39.9 kg, 750 mg; and 40 kg or more, 1250 mg) for 48 weeks in individuals aged 6–19 years and diagnosed with HCLD in Harare, Zimbabwe, and Blantyre, Malawi (BREATHE trial, clinicaltrials.gov identifier NCT02426112). The inclusion criteria for the BREATHE trial were age between 6 and 19 years, perinatally acquired HIV, taking ART for at least six months, having HCLD (defined as forced expiratory volume in 1 s, Z-score < −1.0), no active tuberculosis (TB) or any acute respiratory tract infection. All participants were screened for TB using Xpert MTB/RIF assay upon enrolment. Data collection was performed between June 2016 and September 2019. The detailed study protocol and trial main results are published elsewhere.^13^^,^^24^ Participants were randomised 1:1 using block randomisation stratified by trial site. Participants provided a rectal swab at inclusion (baseline), 12 months (48 weeks), and 18 months (72 weeks) (Fig. 1). Power calculations were done, showing that we needed at least 300 participants to detect a 0.32 standard deviation change in FEV1 with 80% statistical power at a p < 0.05 for the BREATHE main trial.^13^

### Ethical approvals

The study was approved by the London School of Hygiene and Tropical Medicine Ethics Committee (ref. 8818); the Harare Central Hospital Ethics Committee, the Medical Research Council of Zimbabwe (MRCZ/A/1946); College of Medicine Research Ethics Committee Malawi (P.04/15/1719); University of Cape Town Human Research Ethics Committee (754/2015), and Regional Committee for Medical and Health Research Ethics in Norway (REC North) (2015/1650). Clinical trial and importation of drugs were approved by Medicines Control Authority of Zimbabwe (B/279/5/14/2016) and Pharmacy, Medicines and Poisons Board Malawi (PMPB/CTRC/III/76). Written informed consent by guardians and assent by participants aged under 18 years was obtained. Those aged over 18 years gave independent consent. The study was conducted in accordance with the Declaration of Helsinki.

### Data collection

Electronic record forms with filled-in questionnaires were collected on Google Nexus tablets (Google, Mountain View, California, USA) with OpenDataKit software. Paper forms were used for data collection of clinical tests. Data from the paper forms were extracted using CARDIFF 1TELEFORM character optical mark recognition software (version 10.9). Data were managed in a Microsoft Access database (Microsoft, Redmond, Washington, USA).

Rectal swabs were collected by study nurses at each study visit and immediately stored in 1.5 ml of transport medium, PrimeStore MTM (Longhorn Diagnostics, Bethesda, MD). The swabs were stored on ice for a maximum of 1 h before being stored at −80 °C until shipment on dry ice to the laboratory at the University of Cape Town, South Africa. Plasma collected from heparinised blood samples at baseline and 48 weeks was frozen and stored at −80 °C before the analysis. Plasma soluble biomarkers were measured using Luminex multiplex bead assay on a MagPix® instrument according to the manufacturer's protocol (Luminex technology, Hertogenbosch, Netherlands), as previously described.^25^

### Fecal microbiota characterisation

A detailed description of DNA extraction, amplification of the 16S ribosomal ribonucleic acid (rRNA) gene, primers used, library preparation, and sequencing has already been published.^8^ In short, the Zymo Research Quick-DNA Fecal/Soil Microbe Microprep kit (Zymo Research, Irvine, CA) was used for DNA extractions according to the manufacturer's description with minor modifications.^8^ Real-time polymerase chain reaction (RT-PCR) of the 16S rRNA gene was performed to assess DNA quality and total bacterial load, followed by two sets of PCRs targeting the V4 hypervariable region of the 16S rRNA gene. Finally, samples were sequenced on an Illumina Miseq instrument using the Miseq Reagent v3 kit (600 cycles) (Illumina, San Diego, CA).^8^ The data was submitted to ENA (PRJNA1222290).

### Bioinformatics and data filtering

Sequence data were pre-processed and taxonomically classified using the DADA2 pipeline^26^ and the Decontam R package.^27^ Merged sequence read pairs passing default DADA2 quality filtering using the pool=“pseudo”-option were decontaminated using the Decontam R package. Before removing any amplicon sequence variant (ASV) or samples, the data had 6875 unique ASVs. The Decontam outputs showed 264 taxa marked as potential contaminants, which were subsequently removed from the samples included in this study after verification against negative controls from the sequencing.

After the quality control steps, we removed samples with less than 4000 reads (N = 5). Only ASVs classified as bacteria were kept, and ASVs with 0 reads (N = 1361) were removed, leaving 5250 unique ASVs for downstream analysis. For relative abundance analysis, further filtering was done, removing all ASVs with less than ten sequences in total and those appearing in less than two samples (ASV number removed N = 3272). This was done to explore the most relevant ASVs and to reduce noise from very low abundant ASVs. Bacteria were classified at the lowest assigned taxonomic level.

### Statistical analysis

Statistical analyses were performed using R version 4.2.2 (http://www.r-project.org/). Statistical analysis of the microbiota data was done using the phyloseq package^28^ and visualisation by microViz package.^29^ Characteristics between study groups were compared using Student's t-test for continuous parameters and Chi-square test for categorical parameters as the data were normally distributed (visual inspection, Shapiro–Wilk test estimation). Weight-for-age and height-for-age Z-scores (WAZ and HAZ, respectively) were calculated using British 1990 growth reference curves.^30^ Those with Z-scores lower than two were characterised as being underweight and stunted, respectively. α-Diversity was described by richness estimates (observed ASVs and Chao1 index),^31^ whereas the Shannon index was used to describe richness and evenness.^32^ α-Diversity was calculated at a sequencing depth of 4000 reads per sample, which included 99% of the samples. The β-diversity analysis used the *“vegdist”* function in vegan (v.2.5.7)^33^ with PCoA and Bray–Curtis dissimilarity indices. PERMANOVA was conducted with the *“adonis”* function, using 999 permutations.

A linear mixed model (LMM) was fitted using the lme4 and lmerTest package in R^34^ to investigate the effect of AZM on α-diversity. Study sites (Zimbabwe vs. Malawi) were set as a random effect variable. The following fixed effect variables were used: age at enrolment, sex, ever being treated for TB before enrolment, duration of ART by group (6 months–2 years, 2–4 years, 4–6 years, and more than six years), use of cotrimoxazole, season of sampling (dry vs. wet), being stunted (HAZ < −2), and baseline and 48-week values of the relevant index tested. In addition, a model to assess changes over time was applied with the general formula: (selected diversity index) ∼ study arm (AZM or placebo) ∗ timepoint + age of enrolment + sex + previous treatment for TB + duration of ART + co-trimoxazole prophylaxis + season + underweight + trail site + (1 | participant ID). In this model study subjects were treated as random effects while trial site as fixed effects. Plots were made using the ggplot2 package.^35^

**Fig. 2:**
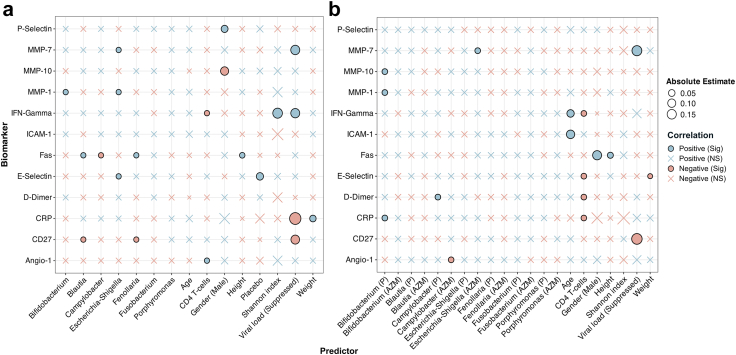
**Associations between systemic biomarkers and various covariates at (a) baseline and (b) after 48 weeks, utilising a linear model (LM).** In panel b, all genera have interacted with the antibiotic in the LM model.

When assessing differences in bacterial relative abundance at the genus level, we chose to use and report results from Aldex2, as this method has shown consistent results across different studies, with high precision and a low false discovery rate (FDR).^36^ p-values were obtained by Wilcoxon test corrected for FDR set to 0.05 by the Benjamini–Hochberg method.^37^ We assessed the correlations among bacterial genera in each cohort (Zimbabwe and Malawi), taking into account AZM treatment and HIV viral load (VL). Since high HIV VL represents poor disease control and may impact gut microbiota,^38^ we decided to divide study participants into two groups (virally suppressed, defined as VL < 1000 copies/ml, and non-suppressed, defined as VL > 1000 copies/ml) at baseline. Spearman's rank correlation was employed to identify significant relationships within detected bacterial genera using the *Hmisc* R package (v.5.1.3). The p-values were adjusted by the Benjamini–Hochberg method that controls for FDR. We visualised only the statistically significant correlations (p < 0.05). Finally, we applied a generalised linear model (LM) to examine the shifts and continuities in the correlations between the top 7 differentially abundant bacterial genera and systemic plasma biomarkers over a period from baseline to 48 weeks using *stats* package in base R as follows: lm(biomarkers ∼ genus ∗ treatment + age + cd4 + sex+ hight + shannon_index + vload + weight + site, data = data). “Biomarkers” and “genus” refer to the same variables shown in Fig. 2. The baseline model was fitted without including treatment interaction.

Before fitting the models, we checked for the assumptions of linearity, normality, homoscedasticity, and independence of residuals using the *performance* (v.012.2)^39^ and *car* R packages (v.3.1.2).^40^ To detect collinearity among the variables, we utilised a variance inflation factor (<5) using *car* R package (v.3.1.2).^40^ The p-values were adjusted by the Benjamini–Hochberg method that controls for FDR.

### Role of the funding source

The funders were not involved in the study design, data collection, data analysis and interpretation of results or writing of this manuscript.

## Results

In total, 347 participants were recruited for the BREATHE trial, of whom 346 were eligible for this study (241 from Zimbabwe and 105 from Malawi). Participants were randomly allocated to AZM (N = 172) or placebo (N = 174). 73.3% of participants in the AZM group and 67.8% in the placebo group completed the 72-week follow-up (Fig. 1). AZM treatment did not significantly improve lung function but was associated with a significantly reduced rate of acute respiratory exacerbations during follow-up.^13^

Participants in the AZM group were slightly younger, with a median age of 14.7 years (interquartile range, IQR 12.6–16.8) vs. 15.8 years (13.0–18.1) in the placebo group (p = 0.03), and more often treated for TB (N = 58 vs. N = 39, p = 0.02). Other characteristics did not differ between groups (Table 1). Between study sites, participants from Malawi were younger (p < 0.001), started ART earlier (p = 0.03), had a lower incidence of reported diarrhoea (p < 0.001), were less frequently treated for TB (p < 0.001), and more often had CD4 T-cell counts < 200 (p < 0.01) compared to participants from Zimbabwe. They also had a higher prevalence of stunting (p = 0.007) and were more often treated with 1st line ART regimen (p = 0.001) compared to participants from Zimbabwe.

**Table 1:** Baseline characteristics.

	Azithromycin (N = 172)	Placebo (N = 174)
Age. median (IQR)	14.7 (12.6–16.8)	15.8 (13.0–18.1)
Sex		
Male. N (%)	93 (54.1%)	84 (48.3%)
Female. N (%)	79 (45.9%)	90 (51.7%)
Study site		
Zimbabwe. N (%)	120 (69.8%)	121 (69.5%)
Malawi. N (%)	52 (30.2%)	53 (30.5%)
Wasted (weight for age Z-score < −2). N (%)	98 (56.9%)	83 (47.7%)
Stunted (height for age Z-score < −2). N (%)	95 (55.2%)	80 (45.9%)
BMI-for-age Z-score. median (IQR)	−1.2 (−1.9 to −0.5)	−1.0 (−1.7 to −0.2)
Episodes of diarrhoea during study period. N (%)	21 (12.2%)	20 (11.5%)
Took co-trimoxazole last three months. N (%)	156 (90.7%)	156 (89.7%)
Took other antibiotics during study period. N (%)	27 (15.7%)	35 (20.1%)
Previously treated for TB. N (%)	58 (33.7%)	39 (22.4%)
FEV1 Z-score. median (IQR)	−1.9 (−2.5 to −1.4)	−1.9 (−2.4 to −1.5)
1st line ART regimen. N (%) (NNRTI-based)	126 (73.3%)	131 (75.3%)
Virally suppressed (VL < 1000 copies/ml). N (%)^a^	99 (57.6%)	94 (54%)
CD4 T-cell count < 200 cells/μl. N (%)	16 (9.3%)	18 (10.3%)
Age at ART initiation. median (IQR)	8.2 (5.0–11.2)	8.9 (6.7–11.6)
Duration of ART (years). Median (IQR)^b^	5.9 (3.8–9.0)	6.4 (3.9–8.2)
Follow-up samples		
12-month (48-week) sample. N (%)	160 (93%)^c^	157 (90.2%)
18-month (72-week) sample. N (%)	128 (74.4%)^d^	118 (67.8%)

Abbreviations: ART, antiretroviral therapy; BMI, body mass index; FEV1 Z-score, forced expiratory volume in 1 s Z-score; IQR, interquartile range; NNRTI, nonnucleoside reverse-transcriptase inhibitor; TB, tuberculosis; VL, viral load.

a Two participants from Malawi, azithromycin group missing baseline HIV viral load.

b Ten participants missing data on duration of ART, one from Zimbabwe in placebo group, nine from Malawi, six in placebo group, three in azithromycin group.

c Three 12-month samples were excluded from downstream diversity analysis due to low read count (<4000).

d Two 18-month samples were excluded from downstream diversity analysis due to low read count (<4000).

### Azithromycin use induces changes in fecal microbiota that persist six months after treatment cessation

There were no statistically significant differences in stool α-diversity between the study groups or sites at baseline. Estimates of total bacterial abundance, as determined by RT-PCR of 16S rRNA bacterial gene, were higher in samples from Zimbabwean participants compared to Malawian (Supplementary Table S1). This was concordant for all three time points.

At 48 weeks, bacterial α-diversity measures (observed ASVs, Chao1 index, and Shannon index) were significantly lower in the AZM group (Table 2). At 72 weeks (six months after cessation of the study drug), there was still a tendency towards lower richness in the AZM group. However, this difference was not statistically significant. Analysis of alpha diversity over time revealed that participants previously treated for TB had significantly lower α-diversity measures (observed ASVs, Chao1 index, and Shannon index) irrespective of study group or time point (Supplementary Table S4).

**Table 2:** Comparison of bacterial α-diversity between study groups at baseline, 48 weeks, and 72 weeks using linear mixed model.

α-Diversity index	Total (N = 895)								
	Baseline (N = 346)			48 weeks (N = 313)			72 weeks (N = 236^c^)		
	AZM (N = 172)	Placebo (N = 174)	p-value^a^	AZM (N = 156)	Placebo (N = 157)	p-value^a^	AZM (N = 120)	Placebo (N = 116)	p-value^a^
	Coefficient (95% CI)			Coefficient (95% CI)			Coefficient (95% CI)		
Observed ASVs	0.36 (−12.67, 13.39)	Ref.	0.958	−23.98 (−37.81, −10.15)	Ref.	<0.001	−7.03 (−21.98, 7.92)	Ref.	0.370
Chao1	0.07 (−13.19, 13.34)	Ref.	0.991	−25.64 (−38.67, −11.61)	Ref.	<0.001	−6.90 (−22.15, 8.34)	Ref.	0.388
Shannon	0.13 (−0.01, 0.26)	Ref.	0.056	−0.21 (−0.35, −0.07)	Ref.	0.004	−0.09 (−0.25, 0.07)	Ref.	0.299
Total bacterial load^b^	−0.13 (−0.55, 0.29)	Ref.	0.547	−0.70 (−1.17, −0.23)	Ref.	0.004	0.08 (−0.41, 0.56)	Ref.	0.745

The placebo group was used as a reference.

Abbreviations: AZM, azithromycin; 95% CI, 95% Confidence Interval; Observed ASVs, observed amplicon sequence variants; Ref., reference.

a Analysis was performed using lmer function from the lme4 package in R version 4.2.2. p-values obtained from the afex package. Fixed effects: Age at enrolment, sex, ever being treated for TB before enrolment, duration of ART by group, use of cotrimoxazole, season of sampling, being stunted (HAZ < −2) and baseline/48 week values of the relevant index tested. Random effect: Study site (Zimbabwe/Malawi).

b 16S copy-number, log-transformed.

c Eight samples at 72 weeks have missing values from 48 weeks and are therefore excluded from analysis (=236 included).

**Fig. 3:**
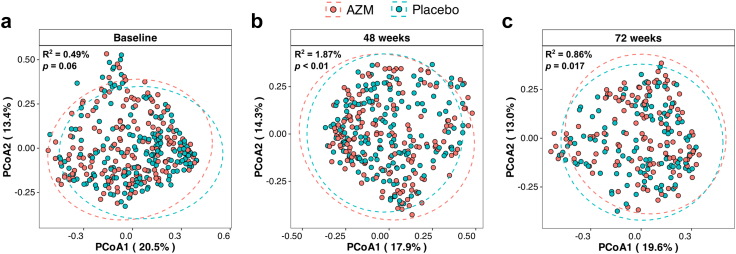
**Principal Coordinate Analysis (PCoA) describing the microbiota composition in the placebo and azithromycin (AZM) groups at (a) inclusion (baseline), (b) 12 months (48 weeks), and (c) 18 months (72 weeks) using Bray–Curtis distance.** The p-value was calculated by adonis2 with 999 permutations.

Next, we investigated changes in the overall composition of microbiota between the study groups. At baseline, β-diversity analysis revealed a borderline significant difference in the bacterial composition between the placebo and AZM groups (PERMANOVA; Bray–Curtis dissimilarity; p = 0.06). However, upon the antibiotic treatment at 48 weeks, there were highly significant differences between the groups (PERMANOVA; Bray–Curtis dissimilarity; p < 0.01). Notably, these differences persisted even after the cessation of antibiotic treatment when assessed at 72 weeks (PERMANOVA; Bray–Curtis dissimilarity; p = 0.017) (Fig. 3).

We identified 30 phyla among all samples analysed, where six bacterial phyla each contributed to more than 1% of the total sequences in the dataset. Specifically, Firmicutes (50.3%), Bacteroidota (32.8%), Actinobacteriota (6.0%), Proteobacteria (5.2%), Campylobacterota (3.4%), and Fusobacteriota (1%) accounted for 98.9% of the bacterial abundance. In total, 24 genera were detected as differentially abundant between rectal swabs from Zimbabwean and Malawian children at baseline (Supplementary Table S2).

On the other hand, we found no differentially abundant bacterial genera between AZM and placebo groups at baseline. A bar plot of relative abundance at the phylum level for all study groups and time points is shown in Supplementary Figure S1. At 48 weeks, 27 genera were detected as differentially abundant between the AZM and placebo groups (Table 3). Participants in the AZM group had a significantly higher abundance of 11 genera and a significantly lower abundance of 16 genera compared to participants in the placebo groups at both trial sites.

**Table 3:** Differentially abundant taxa between azithromycin and placebo group at 48 weeks and 72 weeks.

Phylum	Genus	48 weeks				72 weeks			
		Effect size^a^	Difference (Between)^c^	Pooled std. deviation within each group^c^	p-value^b^	Effect size^a^	Difference (Between)^c^	Pooled std. deviation within each group^c^	p-value^b^
Proteobacteria	Parasutterella	−0.44	−3.17	6.54	<0.001	−0.47	−3.02	6.08	<0.001
Firmicutes	Dorea	−0.26	−1.03	3.05	<0.001				
Firmicutes	Blautia	−0.25	−0.92	2.93	<0.001				
Firmicutes	Flavonifractor	−0.24	−1.42	5.61	0.016				
Firmicutes	Anaerostipesifid	−0.23	−1.45	5.48	0.010				
Actinobacteriota	Eggerthella	−0.22	−1.33	5.34	0.020				
Firmicutes	Fusicatenibacter	−0.22	−1.18	4.48	0.005				
Firmicutes	Lachnoclostridium	−0.22	−1.03	4.03	0.002				
Firmicutes	Lachnospiraceae UCG-004	−0.18	−0.87	4.00	0.012	−0.21	−1.07	4.40	0.031
Firmicutes	Coprococcus	−0.17	−0.86	4.24	0.018				
Firmicutes	[Eubacterium] hallii group	−0.14	−0.71	4.13	0.045				
Fusobacteriota	Fusobacterium	0.21	1.56	7.09	0.026				
Proteobacteria	Escherichia-Shigella	0.21	1.57	7.03	0.020				
Firmicutes	Fenollaria	0.22	1.91	7.98	0.023				
Bacteroidota	Porphyromonas	0.22	1.64	6.93	0.015				
Cyanobacteria	Gastranaerophilales (Order)	0.22	1.33	5.57	0.028				
Firmicutes	Clostridium sensu stricto 1	0.22	1.43	5.81	0.015				
Firmicutes	Christensenellaceae R-7 group	0.23	1.38	5.50	0.008				
Firmicutes	Fastidiosipila	0.25	1.84	6.84	0.011	0.30	2.27	6.84	0.033
Firmicutes	Mitsuokella	0.27	1.72	5.87	0.008				
Firmicutes	Bacilli_RF39 (Order)	0.33	2.05	5.60	<0.001				
Desulfobacterota	Desulfovibrio	0.34	2.19	5.72	<0.001				
Actinobacteriota	Bifidobacterium	0.36	2.71	7.12	<0.001				
Firmicutes	Negativicoccus	0.36	2.72	7.02	<0.001				
Firmicutes	Clostridia UCG-014 (Order)	0.57	3.37	5.42	<0.001				
Campylobacterota	Campylobacter	0.77	5.72	6.87	<0.001	0.35	2.66	6.90	<0.001
Proteobacteria	Sutterella	0.80	5.22	5.72	<0.001	0.51	3.35	5.85	<0.001

Abbreviations: std., standard.

a Negative effect size indicates higher abundance in azithromycin group and positive effect size indicates higher abundance in placebo group.

b Wilcoxon test with FDR set to 0.05 using the Benjamini–Hochberg method.

c Difference (Between) is the difference in mean abundance between groups; Difference (Within) is the pooled standard deviation within each group.

At 72 weeks, five genera remained significantly different between the AZM and placebo groups at both trial sites (Table 3). *Lachnospiraceae UCG-004* and *Parasutterella* were enriched in the AZM group, whereas *Sutterella*, *Campylobacter*, and *Fastidiosipila* were depleted in the AZM group. In the AZM group, four genera were significantly enriched at 72 weeks compared to 48 weeks (Supplementary Table S3). There were no significant differences in relative abundance between 48 and 72 weeks in the placebo group.

### Azithromycin treatment affects gut bacterial community interactions

**Fig. 4:**
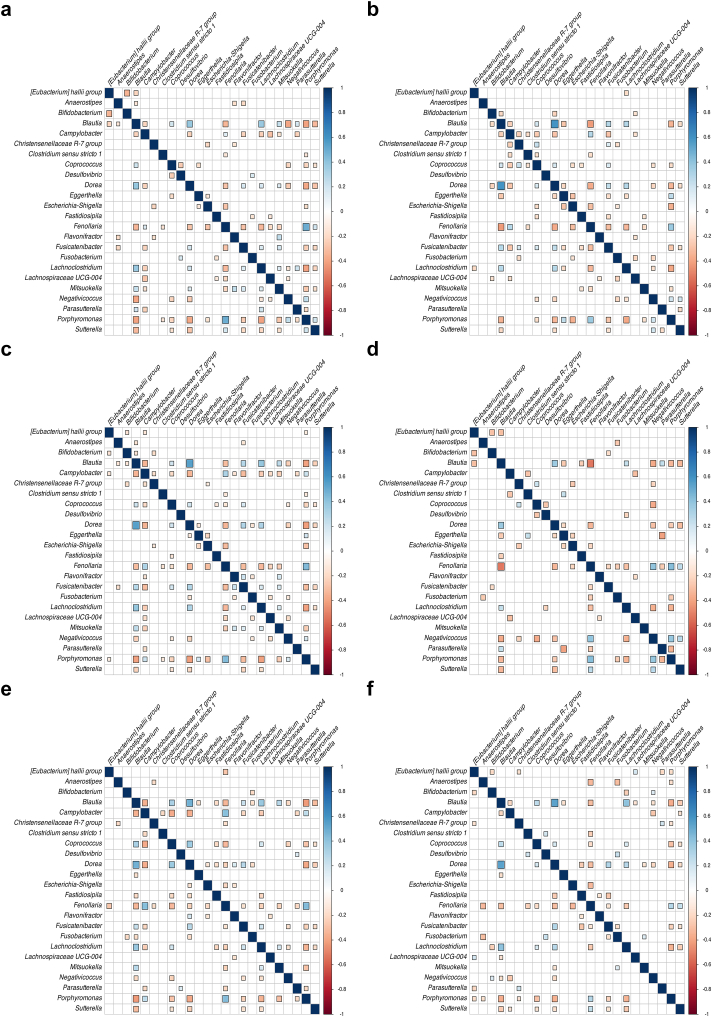
**Spearman correlation matrix plot of relative abundance at the genera level for (a) virally suppressed group (viral load < 1000 copies/ml) at baseline, (b) high viral load group (viral load > 1000 copies/ml) at baseline, (c) Zimbabwean cohort at baseline, (d) Malawian cohort at baseline, (e) placebo group after 48 weeks, and (f) azithromycin-treated group after 48 weeks.** The colour scale represents the Spearman correlation coefficient, and the square size indicates the correlation strength, meaning the absolute value of Spearman coefficient, reflecting the magnitude of the association. All results shown were statistically significant (adjusted p < 0.05).

We also conducted a network analysis of the bacterial community in order to predict the effect of AZM use on bacterial interactions (e.g., positive interactions between bacterial cells such as cross-feeding or negative effects caused by the production of inhibitory molecules). In children who were virally suppressed, the relative abundances of genera such as *Blautia*, *Dorea*, *Fenollaria*, *Lachnoclostridium*, *Negativicoccus*, and *Porphyromonas* exhibited intermediate to strong correlations with each other and with additional genera (Fig. 4a). Conversely, in the non-suppressed group, the strength of these correlations generally diminished. For instance, while no significant correlation was found between *Blautia* and *Negativicoccus*, the correlation between *Dorea* and *Blautia* relative abundances intensified (rho = 0.7, adj. p < 0.05), suggesting that bacterial interactions may be affected by poorly controlled HIV infection reflected by high HIV VL (Fig. 4b). When it comes to trial site, we observed higher interconnectivity (i.e., number of correlations) between bacterial genera in Zimbabwe compared to Malawi, based on the number of significant pairwise correlations observed: 70 in Zimbabwe (Fig. 4c) versus 44 in Malawi (Fig. 4d). However, we acknowledged that Spearman correlations are continuous and reflect various correlation strengths (Supplementary Tables S5a–S5f).

Analysis based on the treatment group revealed notable differences: the placebo group exhibited higher interconnectivity (correlation) between bacterial genera compared to the AZM-treated group after 48 weeks, reflecting the disruptive impact of AZM on microbial interactions (Fig. 4e). Notably, a strong negative correlation between *Dorea* and *Campylobacter* observed in the placebo group (rho = −0.6, adj. p < 0.05), was absent in the AZM group (Fig. 4f). Interestingly, this interconnectivity appeared to be restored six months after the cessation of antibiotic treatment (Supplementary Figure S2).

### Associations between plasma biomarkers and gut microbiome parameters

In the present study, we further investigated associations between plasma biomarkers and gut microbiome composition at baseline, as well as the impact of AZM treatment on these interactions.

We identified a significant positive association between bacterial α-diversity (Shannon index) and IFN-Gamma (LM; t-value = 2.2, adj. p = 0.03) at baseline, which was not observed after 48 weeks in either of the treatment groups (Fig. 2). We also observed a significant positive association between *Bifidobacterium* genus relative abundance and matrix metalloproteinase-1 (MMP-1) at baseline (LM; t-value = 1.85, adj. p = 0.04). This relationship was no longer present after 48 weeks of AZM treatment, while two new positive associations emerged between *Bifidobacterium* and MMP-10, CRP in the placebo group at 48 weeks. The relative abundances of *Blautia* and *Fenollaria* had significant negative association with CD27 (LM; t-value = −2.49, adj. p = 0.01 and t-value = −2.60, adj. p = 0.009, respectively) and a significant positive association with Fas (LM; t-value = 2.26, adj. p = 0.24 and t-value = 2.47, adj. p = 0.016, respectively) at baseline. These relationships were no longer significant at 48 weeks in both study groups. Finally, we found significant positive associations between *Escherichia*-*Shigella* and E-selectin and MMP-1 (LM; t-value = 2.8, adj. p < 0.01 and t-value = 2.1, adj. p = 0.03, respectively) at baseline, which were no longer present after 48 weeks. On the other hand, a significant positive association between *Escherichia*-*Shigella* genera and MMP-7 at baseline (LM; t-value = 1.97, adj. p = 0.047) remained significant only in the AZM group at 48 weeks (LM; t-value = 2.21, adj. p = 0.028).

## Discussion

In the present study, we investigated the effects of AZM treatment on gut microbiota in children and adolescents with HCLD. We found that α-diversity of bacterial taxa, i.e., richness and evenness were significantly lower in the AZM group after 48 weeks of treatment compared to the placebo group. A trend of reduced gut bacterial α-diversity was still present six months after cessation of the drug. In addition, we documented that observed associations between the top seven differentially abundant bacteria and biomarkers at baseline were no longer statistically significant at 48 weeks. Depletion of *Campylobacter* persisted for at least six months after cessation of AZM treatment.

Data on long-term antibiotic effects in HIV-infected population are scarce. However, our results are in line with previous studies investigating the short-term and long-term effects of macrolide antibiotic exposure in both children and adults.^16^^,^^21^^,^^41^, ^42^, ^43^, ^44^ For example, a study of infants aged 6–11 months in India by Parker et al. found that a three-day course of AZM led to reduced α-diversity of the gut microbiota, as measured by operational taxonomic units and Shannon index.^43^ A study in Finnish preschool children by Korpela et al. found macrolides to cause a long-term reduction in the richness of gut microbiota that remained significantly lower up to two years after exposure.^21^ We found that α-diversity returned almost to baseline levels six months after ending AZM treatment. These results may be explained by the fact that participants in our study were older and had more mature gut microbiomes that were more resilient to the disturbances caused by antibiotic use. In addition, the study by Korpela et al. was based on metagenomics and, thus, a different resolution level of the bacterial taxonomy. For future studies, a follow-up beyond 6 months, and use of advanced sequencing methods should be considered.

Another study among healthy pre-school children based on rectal swab samples found α-diversity to be significantly reduced after a 5-day course of AZM treatment, while no difference in α-diversity was observed between those treated with placebo and amoxicillin or cotrimoxazole.^41^ This supports our findings on the effect of macrolides on bacterial α-diversity. Approximately 90 percent of our participants were receiving cotrimoxazole prophylaxis as per WHO guidelines^45^ in both the placebo and AZM groups. In the baseline study of the same participants, we did not find a significant effect of cotrimoxazole on the gut microbiota.^8^ A meta-analysis by McDonnell et al. of antibiotic exposure and gut microbiota in children further supports our findings, as they found that macrolides were associated with an overall reduction in α-diversity and that macrolides reduced richness for twice as long as penicillin.^16^ These results show that long-term use of AZM should be of concern due to the negative effect on gut bacterial α-diversity, especially in people with HIV who are already at increased risk of dysbiotic gut microbiota and enteric infections.^8^^,^^46^^,^^47^

Interestingly, our study revealed that earlier treatment for TB was associated with lower alpha diversity measures across study participants. This is probably explained by these participants receiving broad spectrum antibiotics over time as treatment for TB. While negative impact of TB treatment on gut microbiome diversity has been demonstrated earlier,^48^ our results indicated that these effects persist beyond treatment completion and may potentially compromise gut microbiome homoeostasis.

In line with our findings, Korpela et al. found that macrolide exposure led to a significant increase in *Eggerthella*, *Blautia*, and *Dorea* and a decrease in *Bifidobacterium*.^21^ They also found *Anaerostipes* to remain reduced up to two years after macrolide exposure. In contrast, in our participants, *Anaerostipes* was increased in the AZM group after 48 weeks of treatment, but the difference between AZM and placebo group was not sustained at 72 weeks. Of note, participants in the study by Korpela et al. were younger, not living with HIV, and from a different geographic region than participants in our study. Choo et al. also found *Bifidobacterium* to be depleted and an increase in *Eggerthella* and *Coprococcus* following both AZM and erythromycin exposure among healthy adults.^46^ *Bifidobacterium* is considered to have beneficial traits, including maintaining microbial balance and inhibiting the growth of potential pathogens.^49^ It is most likely highly susceptible to macrolides, which can explain the depletion.^49^ *Eggerthella* has been associated with an increased risk of autoimmune diseases such as ulcerative colitis and an increase in gut microbial translocation.^50^ *Blautia* and *Dorea* can be proinflammatory, and have been associated with inflammatory bowel disease, metabolic disease, and multiple sclerosis.^51^^,^^52^ This reduction in protective commensals and enrichment of proinflammatory taxa in the gut seen after AZM treatment in our cohort could potentially increase systemic immune activation and contribute to persistent chronic inflammation in the HIV-infected population.

Our study demonstrated *Campylobacter* to be significantly depleted after treatment with AZM. *Campylobacter* is known to cause diarrhoeal disease among children, and AZM is a first-line treatment.^53^ Doan et al. investigated the effect of AZM on the gut microbiota in a subset of participants in a large trial of mass distribution of twice-yearly AZM for trachoma control and childhood mortality in Niger, Tanzania, and Malawi.^54^^,^^55^ Although the authors reported no difference in diversity between the AZM and placebo groups, they did report a significant reduction in *Campylobacter*,^54^ which was mirrored in our study. Parker et al. also found *Campylobacter* to be significantly reduced after exposure to AZM.^43^ In a follow-up study, Doan et al. found macrolide resistance determinants to be 7.5 times higher in participants who had received AZM twice yearly for four years compared to those receiving placebo.^22^ The increase in macrolide resistance remained higher six months after the last administration of AZM. In addition, long-term administration of AZM caused an increase in non-macrolide resistance, including beta-lactams, aminoglycosides, trimethoprim, and metronidazole.^22^ This is of concern for future treatment of gastrointestinal *Campylobacter* infections, as the rate of fluoroquinolone resistance among *Campylobacter* species is already high, and macrolide resistance is increasing.^56^

Our study reveals that HIV VL and poorly controlled HIV infection may modulate gut microbiota interactions, either enhancing or diminishing these interactions. This observation aligns with the findings by Chen et al.^57^ The potential consequences of the antibiotic treatment might be a disruption of colonisation resistance conferred by the resident gut microbiota, rendering the host more susceptible to colonisation by potentially pathogenic or drug-resistant bacteria.^54^ Notably, the disrupted interconnectivity appears to be restored six months after cessation of antibiotic treatment. The disruption in the gut microbiome due to antibiotic administration may alter interactions with the immune system and, in turn, contribute to chronic inflammation and immune activation. Such scenario was suggested by Choo JM et al., who found that four weeks of treatment with low dose AZM or erythromycin was accompanied by alterations in systemic biomarkers related to immune homoeostasis.^58^

Treatment with AZM was associated with reduced levels of C-Reactive Protein (CRP), E-Selectin, and MMP-10 in our earlier study of the same cohort.^59^ Here, we explored whether these effects of AZM were mediated by alterations in gut microbiota composition. We did not, however, observe significant correlations between reduced CRP, E-selectin, and MMP-10 levels and top 7 differentially abundant bacterial genera after treatment with AZM. In addition, the majority of the observed associations at baseline disappeared at 48 weeks, irrespective of study group. This finding emphasises the complexity and temporal variability of the gut microbiota in children and youth, which our sequencing strategy might not have fully accessed. Further investigation into these relationships with integration of metagenomic, proteomic and metabolomic data will enhance our understanding of how various factors influence the gut microbiome and its role in host adaptive immune homoeostasis.

Despite significant progress in microbiome research in recent years, a comparison between studies remains a challenge. The differences observed between other studies and ours can be explained by differences in age, geographical location, and trial setting, including the duration of treatment and follow-up. The changes in relative abundance observed were most concordant with studies in other African populations,^41^^,^^44^ highlighting the specificity of geographic location on gut microbiota. The strengths of our study include the randomisation to either AZM or placebo, low loss to follow-up, and the large number of participants compared to other microbiome studies. However, there are several limitations, including the need for more detailed dietary and socioeconomic information and sampling method. All participants included in the study were from urban areas with similar education level, although specific information on this was not collected. While stool samples are more representative of gut microbiome, studies have shown that rectal swabs are an acceptable substitute and are comparable to stool samples for profiling the gut microbiota.^60^^,^^61^

Further, the use of 16S rRNA sequencing only allows for an assessment of relative abundance at the genus level and lacks genomic information on functional properties of detected bacterial taxa, such as the carriage of resistance genes. On the other hand, amplicon sequencing is the method of choice for low biomass samples, with small amounts of DNA, as used in this study. When the settings allow, metagenomic analysis based on sufficient sample material should be considered in future studies.

### Conclusions

We found that long-term AZM treatment in children and adolescents with HCLD reduced richness and evenness of bacterial α-diversity. Most changes in microbial composition after AZM treatment were transient and comparable to those who received placebo six months after cessation of AZM. However, decreased relative abundance of *Campylobacter* in the AZM group persisted for at least six months after cessation of study drug. This may be another positive effect of AZM, added to our findings on reduced rate of acute respiratory exacerbations published previously. Our results confirm previously described differences in diversity and relative abundance after AZM treatment, including an increase in proinflammatory taxa. Further, gut microbial interactions seem to be affected both by HIV treatment status (HIV VL) and antibiotic treatment, but the disruptions in interconnectivity appear to be restored six months after ending the antibiotic treatment.

Future studies should address the development of resistance, especially in potential pathogens such as *Campylobacter*, and the effect of gut dysbiosis on immune activation and its impact on disease progression. The use of comprehensive sequencing methods and longer time of follow-up should be considered. Finally, the benefit of a reduction in the rate of acute respiratory exacerbations among children with HCLD following AZM treatment^13^ needs to be weighed against the disadvantage of reduced gut bacterial diversity and potential increase in antibiotic resistance. Considering this, our findings further suggest that while azithromycin may be appropriate for treatment of active respiratory tract infections, the routine use of long-term azithromycin treatment does not seem to be an optimal preventive strategy in HCLD.

## Contributors

Study conception and design: ES, SCW, RAF, TF, MN. Data collection and management: GM, VS, BREATHE study team. Laboratory work: TTF, SCW, DHB, REA. Bioinformatics: KSM, EH, AB. TTF and ES have accessed and verified the data. Data analysis: TTF, VS, ES, REA, AB, EH. Manuscript preparations and writing: TTF, ES, SCW, REA, VKP, MN. Review: All authors. All authors have read and approved the final version.

## Data sharing statement

The data were submitted to the NCBI sequence read archives (SRA) with the BioProject identification number PRJNA1222290. Link: https://dataview.ncbi.nlm.nih.gov/object/PRJNA1222290?reviewer=rkcbrv5lfjom8c1ski2gfsd156.

## Declaration of interests

The authors declare no competing interests for this manuscript.

## Appendix A Supplementary data

Supplementary Tables (S4–S6)

Caption for Supplementary Materials
