# Supplementary material for: Effect of long-term azithromycin treatment on gut microbial diversity in children and adolescents with HIV-associated chronic lung disease
Source: eBioMedicine. 2025 Jul 5;118:105832. doi: 10.1016/j.ebiom.2025.105832 (PMC12272486; doi:10.1016/j.ebiom.2025.105832)
Supplement: Supplementary Tables (S1–S3) and Figures [file mmc1.docx]

**Effect of Long-Term Azithromycin Treatment on Gut Microbial Diversity in Children and Adolescents with HIV-associated Chronic Lung Disease**

**Supplementary tables and figures**

**Table of Contents**

[Supplementary table 1. α-diversity indices compared between study sites at baseline using generalized linear model (GLM). The Malawi cohort was used as a reference. 2](#_heading=h.gjdgxs)

[Supplementary table 2. Differentially abundant taxa between Zimbabwe and Malawi at baseline. 3](#_heading=h.ff0y84th3od2)

[Supplementary figure 1. Bar plot of relative abundance at phylum level compared between study groups at all three time points sorted by relative abundance of Firmicutes. 4](#_heading=h.30j0zll)

[Supplementary Table 3. Differentially abundant taxa between 48 and 72 weeks in the azithromycin group 5](#_heading=h.zhlio2e84k4h)

[Supplementary figure 2. Spearman correlation matrix plot of relative abundance at the genera level for (a) placebo group after 18 months, and (b) azithromycin-treated group after 18 months. 6](#_heading=h.3dvjlpc8vduk)

# Supplementary table 1. α-diversity indices compared between study sites at baseline using generalized linear model (GLM). The Malawi cohort was used as a reference.

| **α -diversity index** | **Baseline (N = 346)** | |  |
| --- | --- | --- | --- |
|  | **Zimbabwe**  **(N = 241)** | **Malawi**  **(N = 105)** | **p-value** |
|  | **Coefficient (95%CI)*** | |  |
| **Observed ASVs** | 4·04 (-12·24 – 20·32) | Ref. | 0·626 |
| **Chao1** | 4·85(-11·72 – 21·42) | Ref. | 0·565 |
| **Shannon** | -0·09 (-0·25 – 0·07) | Ref. | 0·285 |
| **Total bacterial load (16S copy number, log transformed)** | 1·86(1·34 – 2·39) | Ref. | <0·001 |

*Estimate of coefficient with 95% confidence interval and P values calculated using a linear regression model by the lm-function from the lme4 package in R version 4·2·2 (R studio version 2022·12·0+353).
The model was adjusted for age, sex, history of TB, reported diarrhea, being stunted (HAZ < -2), season of sampling, being on 1^st^ line ART regimen, CD4 T-cell count <200 and age at ART initiation.

Abbreviations: 95% CI, 95% Confidence Interval; Observed ASVs, observed amplicon sequence variants; Ref., reference.

# Supplementary Table 2. Differentially abundant taxa between Zimbabwe and Malawi at baseline.

| **Phylum** | **Genus** | **Effect size*** | **Difference (Between)^†^** | **Pooled std. deviation within each group^†^** | **P-value**** |
| --- | --- | --- | --- | --- | --- |
| Actinobacteriota | Corynebacterium | -0·34 | -3·04 | 8·04 | <0·001 |
| Actinobacteriota | Brachybacterium | -0·36 | -2·56 | 6·44 | <0·001 |
| Actinobacteriota | Brevibacterium | -0·31 | -2·35 | 7·03 | 0·003 |
| Firmicutes | Gallicola | -0·28 | -2·07 | 6·97 | 0·002 |
| Firmicutes | Anaerococcus | -0·31 | -1·86 | 5·27 | <0·001 |
| Actinobacteriota | Actinomyces | -0·26 | -1·74 | 6·28 | 0·018 |
| Firmicutes | Fastidiosipila | -0·24 | -1·67 | 6·79 | 0·013 |
| Actinobacteriota | Kocuria | -0·25 | -1·66 | 5·95 | 0·012 |
| Firmicutes | Clostridium sensu stricto 1 | -0·26 | -1·65 | 5·61 | 0·005 |
| Proteobacteria | Acinetobacter | -0·23 | -1·61 | 5·93 | 0·026 |
| Firmicutes | Ezakiella | -0·22 | -1·57 | 6·39 | 0·009 |
| Firmicutes | Peptococcus | -0·25 | -1·53 | 5·58 | 0·005 |
| Firmicutes | Helcococcus | -0·21 | -1·48 | 6·20 | 0·036 |
| Firmicutes | Finegoldia | -0·20 | -1·37 | 6·13 | 0·042 |
| Firmicutes | Murdochiella | -0·20 | -1·35 | 6·22 | 0·020 |
| Firmicutes | Holdemanella | -0·21 | -1·34 | 5·96 | 0·018 |
| Firmicutes | Lachnospiraceae (Family) | 0·22 | 0·86 | 3·38 | 0·003 |
| Firmicutes | Lachnospiraceae UCG-004 | 0·17 | 0·90 | 4·69 | 0·03 |
| Firmicutes | Dialister | 0·21 | 1·26 | 5·15 | 0·006 |
| Proteobacteria | Sutterella | 0·27 | 1·53 | 5·37 | <0·001 |
| Bacteroidota | Prevotella | 0·34 | 1·57 | 4·18 | <0·001 |
| Firmicutes | Lachnoclostridium | 0·33 | 1·66 | 4·33 | <0·001 |
| Bacteroidota | Parabacteroides | 0·41 | 2·31 | 4·93 | <0·001 |
| Bacteroidota | Bacteroides | 0·39 | 2·50 | 5·72 | <0·001 |
| Campylobacterota | Campylobacter | 0·36 | 2·66 | 6·76 | <0·001 |

* Negative effect size indicates higher abundance in Malawi and positive effect size indicates higher abundance in Zimbabwe.
** Wilcoxon test with FDR set to 0·05 using the Benjamini-Hochberg method

**^†^** Difference (Between) is the difference in mean abundance between groups; Difference (Within) is the pooled standard deviation within each group.

Abbreviations: std., standard


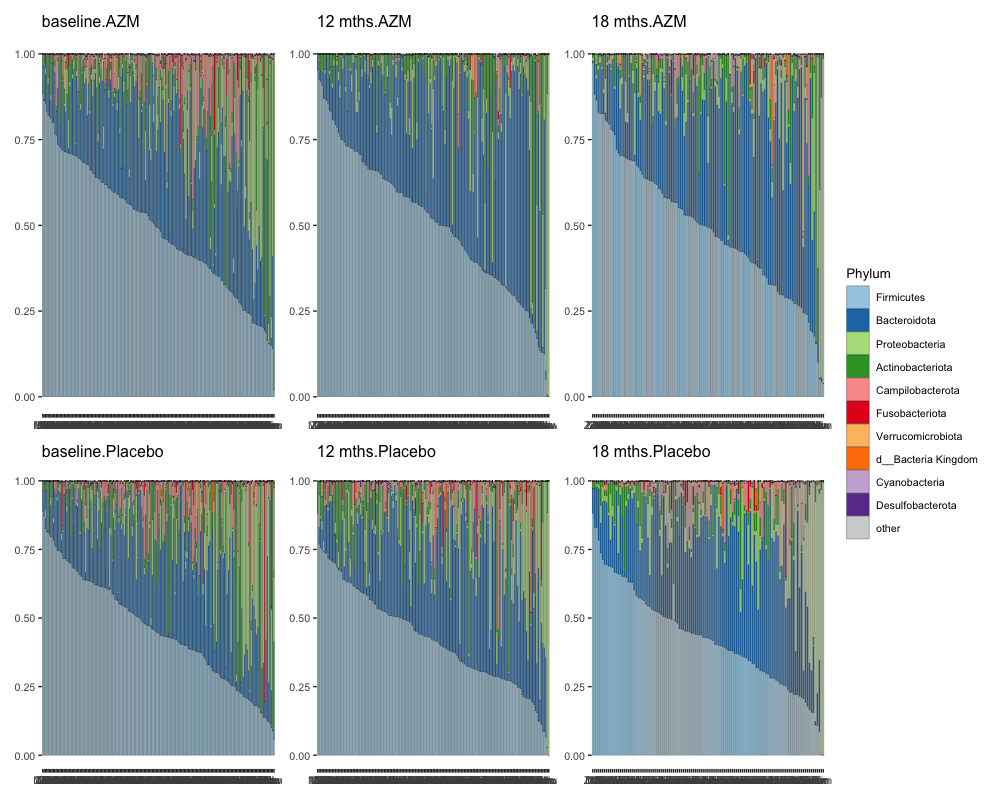
 **Supplementary figure 1. Bar plot of relative abundance at phylum level sorted by relative abundance of Firmicutes, compared between study groups at baseline, 48 weeks (12 months) and 72 weeks (18 months).**

X-axis; sample ID, one bar per sample. Y-axis; relative abundance of specific bacteria at phylum level

# Supplementary Table 3. Differentially abundant taxa between 48 and 72 weeks in the azithromycin group

| **Phylum** | **Genus** | **Effect size*** | **Difference (Between)^†^** | **Pooled std. deviation within each group** **^†^** | **P-value*** |
| --- | --- | --- | --- | --- | --- |
| Proteobacteria | Escherichia-Shigella | 0·26 | 1·99 | 7·00 | 0·028 |
| Firmicutes | Negativicoccus | 0·28 | 2·16 | 6·94 | 0·018 |
| Firmicutes | Clostridia UCG-014 (Order) | 0·34 | 2·17 | 5·75 | 0·006 |
| Campylobacterota | Campylobacter | 0·45 | 3·57 | 7·14 | <0·001 |

* Negative effect size indicates higher abundance at 48 weeks and positive effect size indicates higher abundance at 72 weeks.
** Wilcoxon test with FDR set to 0·05 using the Benjamini-Hochberg method

**^†^** Difference (Between) is the difference in mean abundance between groups; Difference (Within) is the pooled standard deviation within each group.

Abbreviations: std., standard

**Supplementary figure 2. Spearman correlation matrix plot of relative abundance at the genera level for (a) placebo group after 72 weeks, and (b) azithromycin-treated group after 72 weeks.**
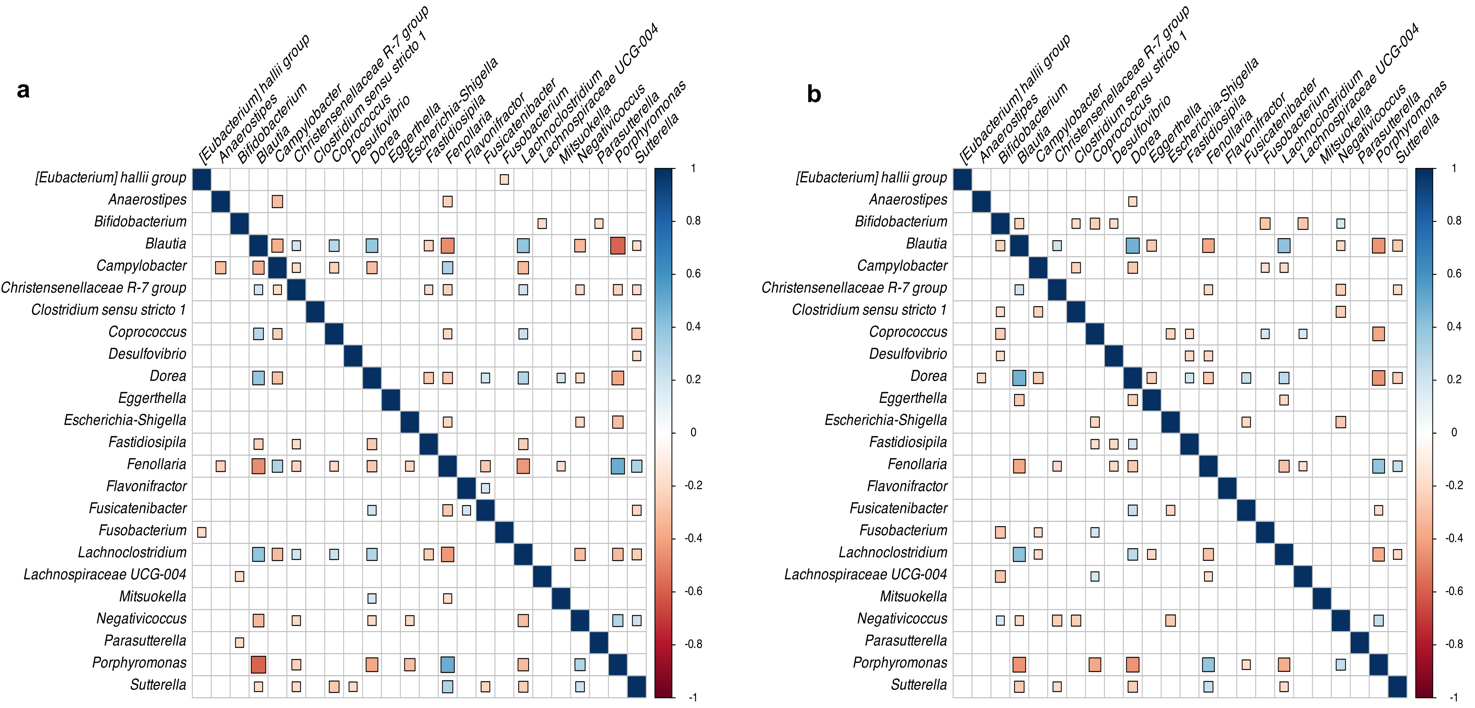


The colour scale represents the Spearman correlation coefficient, and the square size indicates the correlation strength, meaning the absolute value of Spearman coefficient, reflecting the magnitude of the association. All results shown were statistically significant.
