# Supplementary material for: Effect of long-term azithromycin treatment on gut microbial diversity in children and adolescents with HIV-associated chronic lung disease
Source: eBioMedicine. 2025 Jul 5;118:105832. doi: 10.1016/j.ebiom.2025.105832 (PMC12272486; doi:10.1016/j.ebiom.2025.105832)
Supplement: Caption for Supplementary Materials [file mmc3.docx]

**Effect of Long-Term Azithromycin Treatment on Gut Microbial Diversity in Children and Adolescents with HIV-associated Chronic Lung Disease**

**Caption for supplementary material**

**Supplementary table 1**. α-diversity indices compared between study sites at baseline using generalized linear model (GLM). The Malawi cohort was used as a reference.

**Supplementary table 2**. Differentially abundant taxa between Zimbabwe and Malawi at baseline.

**Supplementary figure 1.** Bar plot of relative abundance at phylum level sorted by relative abundance of Firmicutes, compared between study groups at baseline, 48 weeks (12 months) and 72 weeks (18 months).

**Supplementary Table 3.** Differentially abundant taxa between 48 and 72 weeks in the azithromycin group.

**Supplementary figure 2.** Spearman correlation matrix plot of relative abundance at the genera level for (a) placebo group after 72 weeks, and (b) azithromycin-treated group after 72 weeks.

**Supplementary table 4.** Changes in alpha diversity measures over time using linear mixed effect models.

**Supplementary table 5a**. Spearman’s rank correlation between bacterial genera for virally suppressed group (VL<1000 copies/ml) at baseline.

**Supplementary table 5b.** Spearman’s rank correlation between bacterial genera for high viral load group (VL>1000 copies/ml) at baseline.

**Supplementary table 5c**. Spearman’s rank correlation between bacterial genera for Zimbabwean cohort at baseline.

**Supplementary table 5d.** Spearman’s rank correlation between bacterial genera for Malawian cohort at baseline.

**Supplementary table 5e.** Spearman’s rank correlation between bacterial genera for placebo group after 48 weeks.

**Supplementary table 5f.** Spearman’s rank correlation between bacterial genera for azithromycin-treated group after 48 weeks.

**Supplementary table 6a.** Associations between systemic biomarkers and various covariates at baseline using a linear model.

**Supplementary table 6b.** Associations between systemic biomarkers and various covariates after 48 weeks using a linear model.
